# Clinical and Molecular Epidemiology of *Stenotrophomonas maltophilia* in Pediatric Patients From a Chinese Teaching Hospital

**DOI:** 10.3389/fcimb.2020.00411

**Published:** 2020-08-11

**Authors:** Zhongliang Duan, Juanxiu Qin, Cui Li, Chunmei Ying

**Affiliations:** ^1^Department of Clinical Laboratory, Obstetrics and Gynecology Hospital of Fudan University, Shanghai, China; ^2^Department of Clinical Laboratory, Renji Hospital Affiliated to Shanghai Jiaotong University School of Medicine, Shanghai, China

**Keywords:** *Stenotrophomonas maltophilia*, MLST, PFGE, virulence gene, biofilm

## Abstract

**Objective:** To study the molecular epidemiological characteristics of *Stenotrophomonas maltophilia* (SMA) isolated from patients in a pediatric teaching hospital in Shanghai so as to provide data for the prevention and treatment of SMA.

**Methods:** Non-repetitive SMA strains were isolated from patients from January 2013 to December 2014. The cloning characteristics were analyzed using multilocus sequence typing (MLST) and pulsed field gel electrophoresis (PFGE), and the drug resistance was determined using the Kirby-Bauer disk method. Virulence genes and biofilm genes were detected using polymerase chain reaction (PCR). The biofilm forming ability was analyzed using the semi-quantitative biofilm formation test.

**Results:** A total of 104 strains were collected, primarily from the pediatric intensive care unit and thoracic surgery, and these strains were isolated from sputum sources (*n* = 82). A majority of the patients were male (67/104), and the age range was between 6 days and 12 years old. A total of 95 patients had 1–3 baseline diseases. All of the patients had prior use of 1–4 antimicrobial agents. A total of 59 STs were detected using the MLST analysis, of which 45 were new. The sequence types of the SMA were scattered, with no trend in the clonal spread. The PFGE showed that the 104 strains could be divided into 93 clusters, with no obvious cluster aggregations. All of the strains were susceptible to levofloxacin, trimethoprim/sulfamethoxazole, and minocycline. The positive rates of the virulence genes *stmPr1, stmPr2, smf-1*, and *smlt3773 locus* were 98.1, 86.5, 100, and 91.3%, respectively. All of the strains had biofilm formation, and most of the strains had strong biofilm formation abilities. The positive rates of the three biofilm genes *rmlA, spgM*, and *rpfF* were 83.7, 100, and 45.2%, respectively. However, the point mutations of *rmlA* and *spgM* with strong biofilm formation abilities were significantly different from those with weak biofilm formation abilities.

**Conclusion:** Most infected patients had prior use of antibiotics and underlying diseases, and the positive rate of the virulence gene was high. The strains were susceptible to three kinds of antibiotics and had strong biofilm formation abilities. The mutations of *rmlA* and *spgM* may be related to the biofilm formation ability, and no obvious clonal transmissions were found in the same clinical department.

## Introduction

*Stenotrophomonas maltophilia* (SMA) is a type of non-fermenting Gram-negative bacilli that widely exists in the environment. Currently, most reports of SMA infection have examined adult patients, but the description of childhood infections are fewer. SMA is an opportunistic pathogen that colonizes in human beings, and it produces two chromosomal beta-lactamases, L1 and L2, which make SMA intrinsically resistant to many β-lactam antibiotics (Okazaki and Avison, 2008). In particular, it is nearly 100% resistant to imipenem and other carbapenem drugs. In addition, it can also have a certain degree of resistance to aminoglycosides with the help of aminoglycoside-modifying enzymes and resistance to some antibiotics due to efflux pump expression (Samonis et al., 2012), which causes significant difficulty for the use of drugs in clinic, especially in patients with low immunity.

The immune systems of children are weak, which makes clinical infection in children increase and also makes clinical treatment difficult. With the abuse of antibiotics, immunosuppressors, and the wide use of invasive medical equipment, the nosocomial infection rate of SMA has gradually increased (Brooke, 2012). Therefore, in this study, the clone spread of SMA by PFGE and MLST is examined to compare with other studies conducted both in China and abroad. The drug resistance rates of SMA in the different areas were found to be quite different. For example, Hu et al. (2018) found that the resistance to trimethoprim/sulfamethoxazole (TMP/SMX) increased from 29.7 to 47.1% during 10 years of surveillance in China. Haowa Madi (Madi et al., 2016) reported that children in a tertiary children's hospital were all susceptible to ciprofloxacin, chloramphenicol, tetracycline, and levofloxacin. One aim of this study is to update the status of drug resistance in Shanghai.

The virulence factors for SMA, such as extracellular enzymes, proteases, and esterase, can cause damage to human beings. The genes *stmPr1, stmPr2, smf-1*, and *smlt3773 locus* take part in encoding these virulence factors (Nicoletti et al., 2011). The biofilm can make SMA adhere to surfaces of abiotic materials and human tissues. The biofilm genes (*rmlA, spgM*, and *rpfF*) have been reported to be associated with biofilm formation, and their mutations have been shown to be different in both strong and weak biofilm formation strains in adult patients (Zhuo et al., 2014). Up to now, virulence genes and biofilm genes in pediatric patients have been poorly characterized.

The surveillance of pediatric SMA infection in China requires more attention. In addition, there are great differences in drug resistance rates and other characteristics between pediatric specialized hospitals and the pediatric wards of general hospitals. Therefore, this study aims to provide data for the clinical prevalence of SMA infection in a pediatric specialized hospital.

## Materials and Methods

### Strains

Non-repetitive strains of SMA were isolated from inpatients in Shanghai Children's Medical Center from January 2013 to December 2014. These strains were isolated from different wards and different parts of body. They were then cultured in blood agar overnight at 37°C and identified using MALDI-TOFMS.

### Primary Reagents and Instruments

The DNA extraction kit and protease K were purchased from Tiangen Biochemistry Technology Co., Ltd., and the crystal violet and the phosphate buffer solution (PBS) buffer were purchased from Shanghai Shenggong Bioengineering Co., Ltd. The PCR polymerase, restriction endonuclease, and DNA marker were purchased from TaKaRa Company of Japan, and the MALDI-TOFMS mass spectrometer was purchased from Bruker Daltonik Company (Germany). The PCR instrument was purchased from the Applied Biosystems Company of the United States, and the enzyme labeling instrument was purchased from the Biotek Company of the United States. The pulsed gel electrophoresis system was purchased from Bio-Rad Company, the Ultra Micro Spectrophotometer (NanoDrop2000) was purchased from Thermo Company, and the Ultraviolet Gel Imager was purchased from Shanghai Tianneng Company. The −80°C ultra-low temperature refrigerator and incubator were purchased from Thermo Company, while the micro-pipette and centrifuge were purchased from Eppendorf Company (Germany).

### Antibiotic Susceptibility Test

According to the CLSI 2019 standard, three types of antibiotics (levofloxacin, TMP/SMX, and minocycline) were tested using the Kirby-Bauer disk diffusion method.

### Multilocus Sequence Typing (MLST)

The website http://pubmlst.org/smaltophilia/ was referenced to synthesize seven pairs of housekeeper gene primers (Brooke, 2012). The sequence was analyzed using the DNAstar software and submitted to the MLST database to obtain the ST type. Seven pairs of housekeeper genes were spliced and constructed using the MEGA 4 software for evolutionary analysis.

### Pulsed Field Gel Electrophoresis (PFGE)

According to the findings of Tanimoto's and Shueh CS's (Shueh et al., 2013; Tanimoto, 2013) and the preliminary experiments of this study, PFGE was performed using the BioRad system. The *Salmonella* serotype Braenderup strain (H9812) was used as the molecular weight marker. The PFGE profiles were calculated using BioNumerics 4.0 (Applied Maths, Belgium).

### Virulence Gene Detection

After the strains were cultured in trypticase soy broth (TSB, Difco-Becton Dickinson, NJ, USA) overnight at 37°C, the DNA was extracted from the SMA strains using a Genomic DNA Purification Kit. The obtained DNA was identified with the virulence genes using multiplex PCR. The primers for the virulence genes (*stmPr1, stmPr2, smf-1*, and *smlt3773 locus*) were synthesized according to Nicoletti's protocol, as shown in Table 1. The PCR products were detected using electrophoresis on agarose gels and then stained using 0.5 μg/mL of ethidium bromide. The positive rate of the virulence genes was calculated according to the visualization results.

**Table 1:** Primers for the MLST and detection of biofilm and virulence genes.

**Primer**	**Gene**	**Sequence(5′-3′)**	**Size(bp)**
rmlA-F	*rmlA*	CGGAAAAGCAGAACATCG	1222
rmlA-R		GCAACTTGGTTTCAATCACTT	
spgM-F	*spgM*	ATACCGGGGTGCGTTGAC	2750
spgM-R		CATCTGCATGTGGATCTCGT	
rpfF-F	*rpfF*	CACGACAGTACAGGGGACC	1140
rpfF-R		GGCAGGAATGCGTTGG	
stmPr1-F	*stmPr1*	TGGATGCTTGGTCCCGTAGT	2540
stmPr1-R		CCGTGGTGTCGGCTTCGATCTCT	
stmPr2-F	*stmPr2*	GCCGATTCCGGCATTCACACC	1764
stmPr2-R		GGTCAGGCCCGAGAAGGTGCT	
smf-1-F	*smf-1*	GGAAGGTATGTCCGAGTCCG	674
smf-1-R		GCGGGTACGGCTACGATCAGTT	
smlt3773 locus-F	*smlt3773 locus*	CGGTGCCGAACTCGTAACCGG	1342
smlt3773 locus-R		CTTCCGGCCATGGCAGGCGAA	

### Biofilm Gene Analysis

According to Zhou's methods, the biofilm genes (*rmlA, spgM*, and *rpfF*) were amplified and sent to Shanghai Platinum Co., Ltd. for sequencing. SeqMan software was used to compare the point mutations among the strains with different biofilm formation intensities.

### Biofilm Formation Assay

The biofilm formation ability was detected using a crystal violet staining assay. The *S. maltophilia* was overnight cultured, diluted in TSB to D_600_ of 0.01, and taken to a 200 μl the dilution and then placed in a 96-well plate (Corning, USA) at 37°C for 24 h. The cultures were then fixed at 60°C for 1 h, washed with sterile PBS four times, 50 μL crystal violet dye was added into each well for 5 min, and then washed using running tap water. Finally, the plates were dried at room temperature, and 250 μL of 33% glacial acetic acid was added to each well to dissolve the staining for 15 min. Then the optical density (OD) was measured at 492 nm. The cut-off (ODc) was defined using the 3× the standard deviation above the mean OD of the control wells, and the biofilm formation ability was classified as follows: no biofilm (OD≤ODc), weak biofilm (ODc<OD≤2×ODc), moderate biofilm (2×ODc<OD≤4×ODc), and strong biofilm (4×ODc<OD).

### Statistical Analysis

The statistical analysis was conducted using SPSS19 and Prism5 software. The normality was calculated using the Shapiro-Wilk test. The Chi-square test was analyzed for the categorical data. Independent-sample *t*-tests or a One-Way ANOVA were applied for the continuous variables. The positive rate was expressed using a percentage (%). The median and range or Mean ± SD was used for the continuous variables. The correlation was compared using the Pearson correlation coefficient. The difference was considered statistically significant when *P* < 0.05.

## Result

### Clinical Information of Patients and Strains

**Figure 1:**
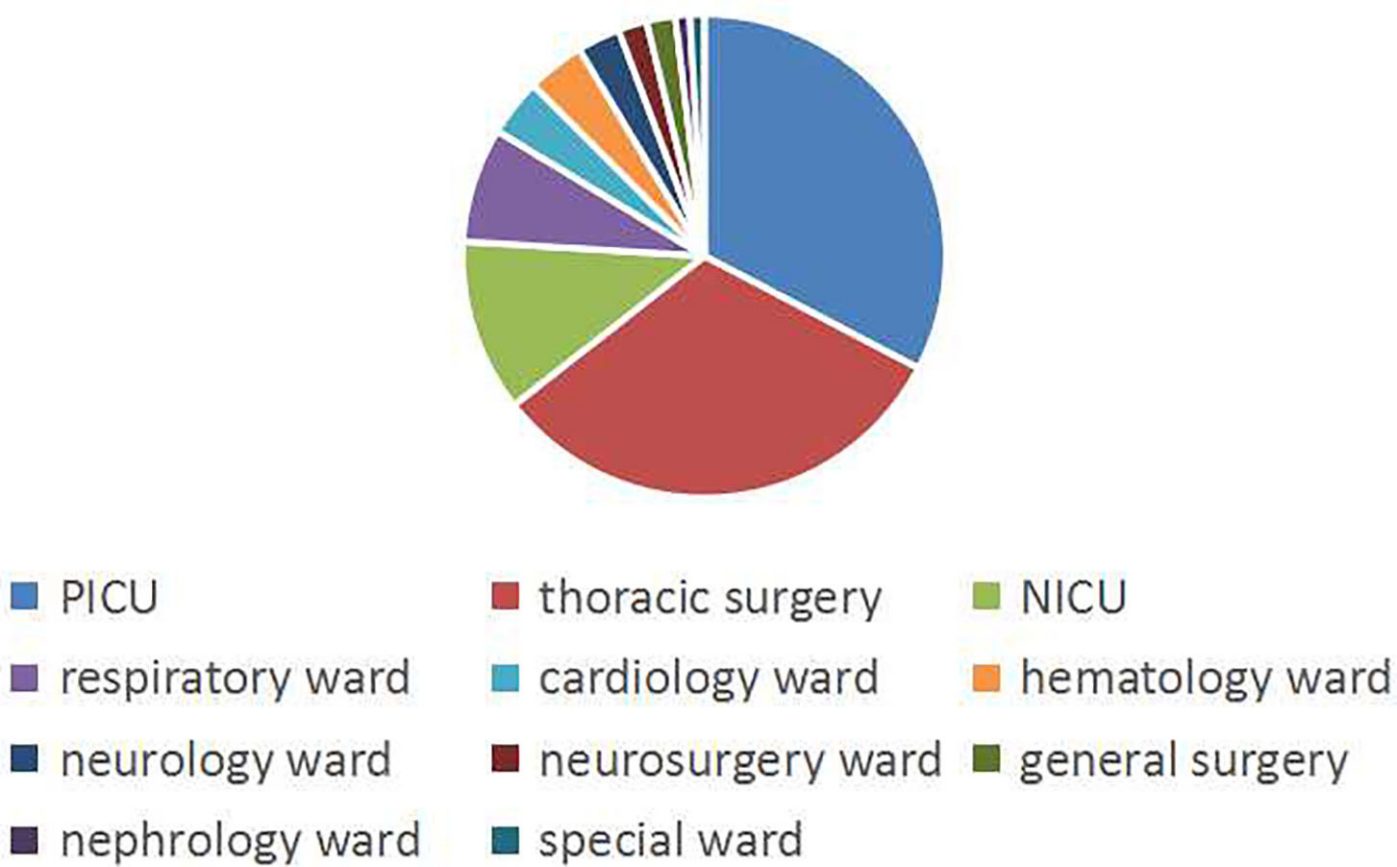
The ward distribution of SMA infections. The pie graph shows the distribution of the wards. Blue, red, and yellow represent the most isolated wards, PICU, thoracic surgery, and NICU, respectively.

**Figure 2:**
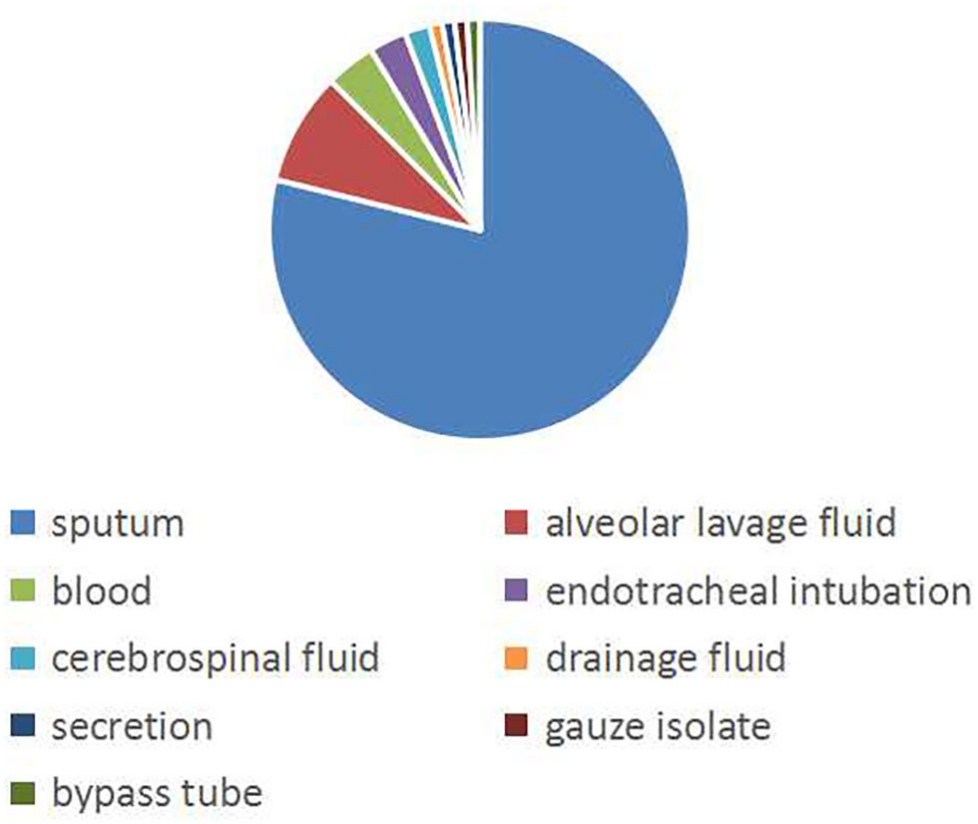
The isolation source of the specimen. The pie graph shows the source of the strains. Blue represents the majority of strains isolated from sputum.

A total of 104 non-repeating SMA strains were collected from January 2013 to December 2014. The ward distribution is shown in Figure 1. The most isolated strains were from the pediatric intensive care unit (PICU,34 strains). There were 33 strains in thoracic surgery, 12 strains in the neonatal intensive care unit (NICU), 8 strains in the respiratory ward, 4 strains in the cardiology ward, 4 strains in the hematology ward, 3 strains in the neurology ward, 2 strains in the neurosurgery ward, 2 strains in general surgery, 1 strain in the nephrology ward, and 1 strain in the special ward. Among the patients, 67 (64.4%) were males and 37 (35.6%) were females. The age of the patients ranged from 6 days to 12 years old, of which 88 (84.6%) were less than or equal to 1 year old, 10 (9.6%) were between 1 year old and 3 years old, and 6 were more than 3 years old (5.8%). A total of 50 patients (48.1%) had invasive examinations or treatments. For the source of the specimens, there were 82 cases from sputum, 9 cases from alveolar lavage fluid, 4 cases from blood, 3 cases from endotracheal intubation, 2 cases from the cerebrospinal fluid, 1 case from drainage fluid, 1 case from secretions, 1 case from gauze isolate, and 1 case from the bypass tube, as shown in Figure 2.

### Prior Drug Use and Baseline Diseases

A total of 95 of the patients had 1–3 types of underlying diseases, including 58 with pneumonia or pulmonary infection, 50 with congenital heart disease or cardiac insufficiency, three with leukemia and others (some patients have multiple diseases). A total of nine patients had no basic diseases, and there were primarily premature infants, asphyxia, and other factors in the hospital.

All of the patients had prior antibiotic usage (1–4 types), of which 31 used at least 3 types, primarily including carbapenem (85/104) and cephalosporins (80/104), cephalosporins (50/104), penicillins (48/104), glycopeptides (45/104), aminoglycosides (14/104), and macrolides (13/104). The results are shown in Table 2.

**Table 2:** The clinical characteristics of the pediatric patients.

	**Pediatric (*n*=104)**
**Demographics**	
Age(year, median,range)	0.7 (6 days-12)
Gender: male	67 (66.4%)
**Baseline diseases, n(%)**	
Hypertension	0
Heart Disease	50 (48.1)
Malignancy	1 (1)
Pulmonary Disease	58 (55.8)
Liver Disease	1 (1)
Leukemia	3 (2.9)
Head trauma	3 (2.9)
**Strain isolation n(%)**	
ICU	46 (44.2)
Sputum	82 (78.9)
**Invasive operation n(%)**	50 (48.1)
**Previous antibiotics usage n(%)**	
The number of antibiotics ≥3	31 (29.8)
Cephalosporins	80 (76.9)
Carbapenems	85 (81.7)
Enzyme Inhibitors	72 (69.2)
Quinolones	0
Glycopeptides	45 (43.3)
Aminoglycosides	14 (13.5)

### MLST Clonal Typing

The clonal typing infected with SMA was also relatively scattered and could be divided into 59 ST types. Among them, 45 types were different from the known types in Pubmed, and they were new ST types. These were submitted to the Pubmed system, and new ST names were obtained for them.

**Figure 3:**
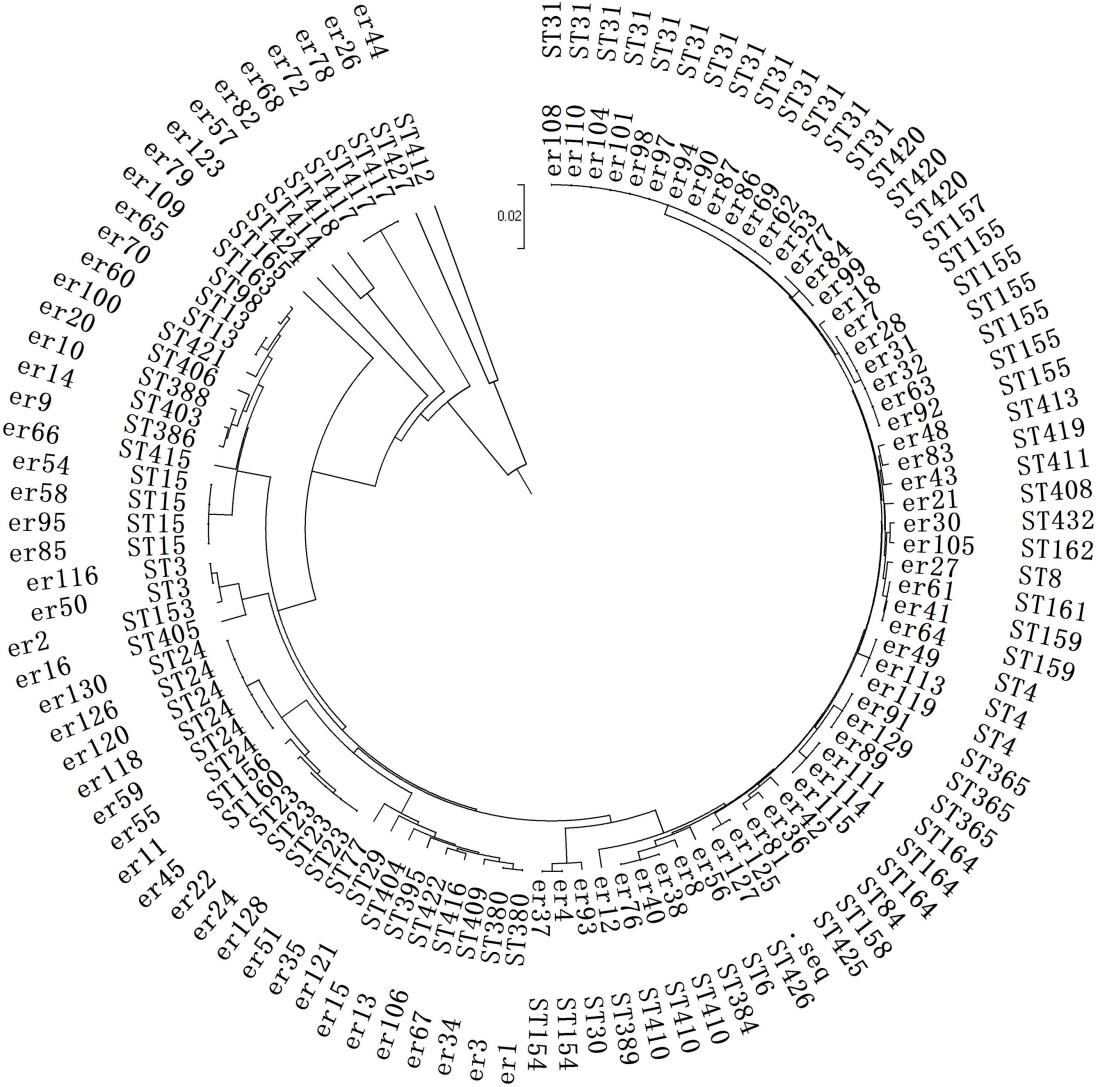
Phylogenetic analysis of the MLST in the SMA strains. This is a neighbor-joining tree analysis for the concatenated data for all 7 loci of the 104 SMA strains. The tree was rooted with the corresponding concatenate.

The primary ST type was ST31 from 13 strains (not in the same department, but distributed in five different wards). The rest were scattered, including ST3 (2 strains), ST4 (3 strains), ST6 (1 strain), ST8 (1 strain), ST13 (2 strains), ST15 (4 strains), ST23 (4 strains), ST29 (1 strain), ST30 (1 strain), ST24 (6 strains), ST77 (1 strain), ST84 (1 strain), and ST98 (1 strain), The detailed results are shown in Figure 3.

A total of 34 strains from the child intensive care unit (the most ward) were classified into 22 different ST types. There was no obvious clonal transmission, and no identical ST cross-infection was found in the other departments.

### PFGE Typing Results

According to the fragment diagnostic criteria of the PFGE typing, strains with no more than three bands of difference can be classified into a group or cluster (Tenover et al., 1995; Valdezate et al., 2004). The results showed that these strains could be divided into 93 clusters, among which, 4 strains (er108, er110, er97, and er101) could be divided into the same cluster, but not from the same department. The other strains were distant, which could not be divided into the same cluster. These results suggest that there was no outbreak in the department. The detailed results are shown in the Supplement Figure 1.

### Virulence Gene Detection

The positive carrier rates of *stmPr1, stmPr2, smf-1*, and *smlt3773 locus* were 98.1% (102/104), 86.5% (90/104), 100 (104/104), and 91.3% (95/104), respectively. Among them, 80 strains carried all 4 of these genes.

### Drug Resistance Analysis

A total of 104 strains of SMA were all susceptible to 3 targeted drugs (levofloxacin, TMP/SMX, and minocycline).

### Biofilm Formation

**Figure 4:**
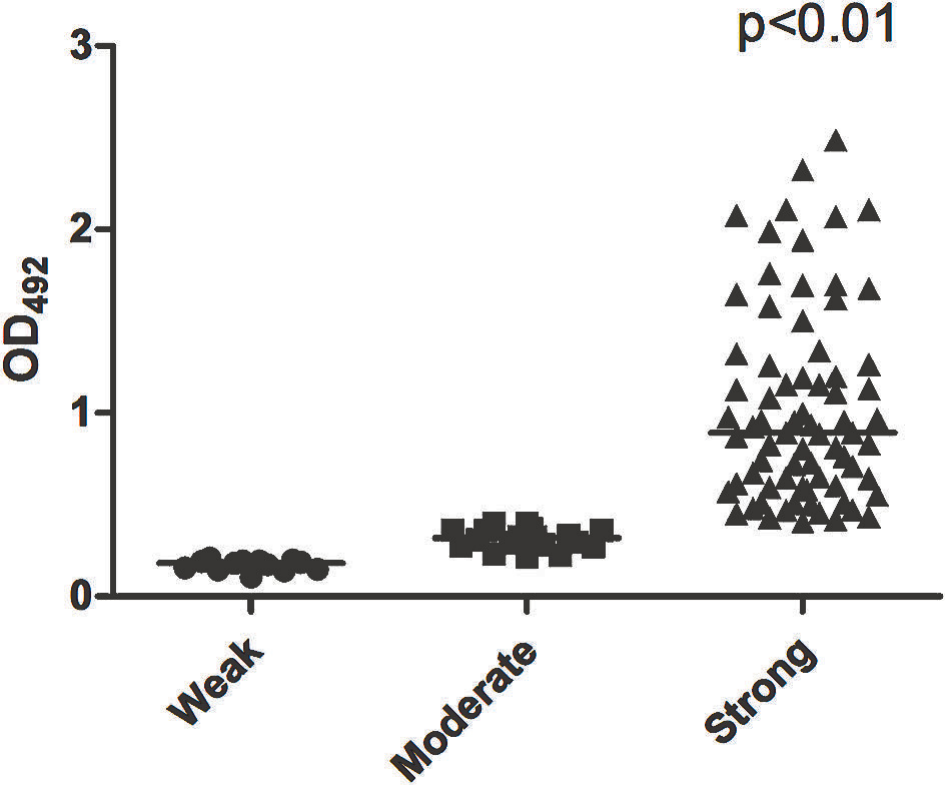
Scattergram illustrating the ability of biofilm formation. The number of strains forming strong biofilms is significantly greater than the weak and moderate number of strains.

**Figure 5:**
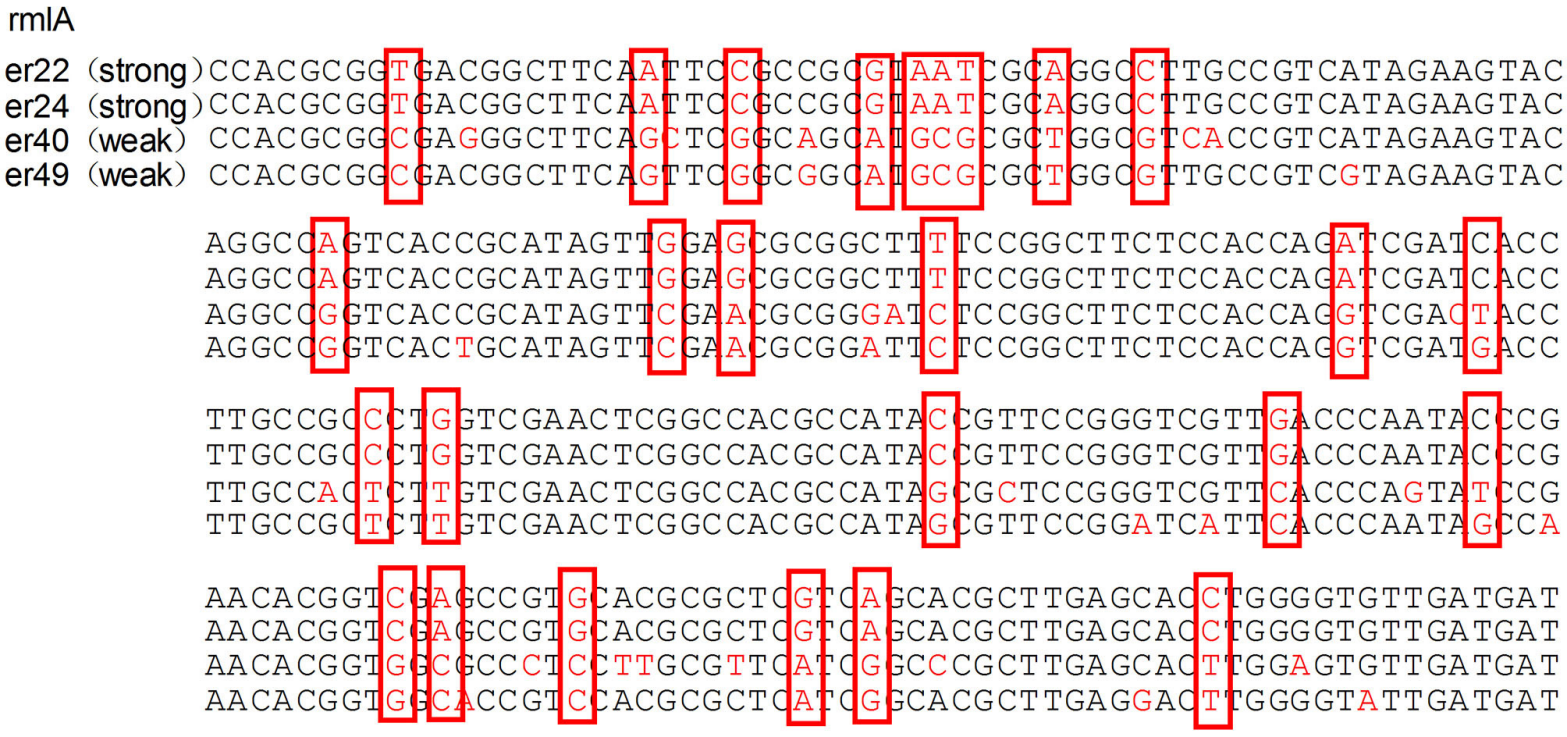
Analysis of the rmlA gene mutation in strains with different biofilm formation abilities. The mutations of the rmlA gene in strains with strong biofilm formation abilities are significantly different from the weak ones. The portions of the DNA bases are shown in the red box.

**Figure 6:**
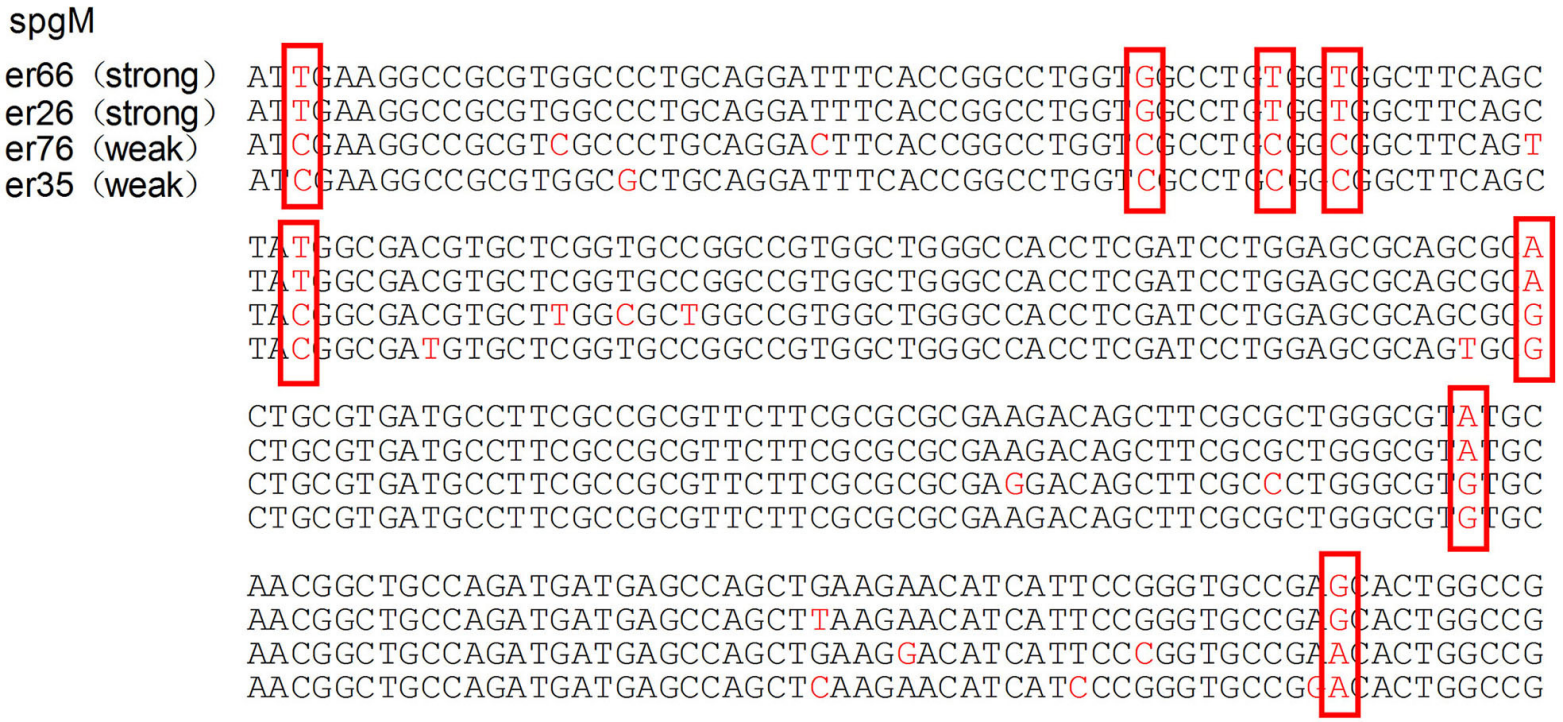
Analysis of the spgM gene mutation in strains with different biofilm formation abilities. The mutations of the spgM gene in strains with strong biofilm formation abilities are significantly different from the weak ones. The portions of the DNA bases are shown in the red box.

The median biofilm formation ability of the SMA was OD_492_ =0.80 (range 0.11–2.49). A total of 13 strains formed weak biofilms, 17 strains formed moderate biofilms, and 74 strains had strong biofilm formation abilities, as shown in Figure 4. There was also no significant difference between the different wards. The positive rates of the three biofilm genes *rmlA, spgM*, and *rpfF* were 83.7% (87/104), 100% (104/104), and 45.2% (47/104). However, the point mutations of the *rmlA* and *spgM* genes in the strains with strong biofilm formation abilities were significantly different from those with weak biofilm formation abilities, as shown in Figures 5, 6. According to the statistical analysis, there was no correlation between the strain virulence genes and the biofilms, as shown in Supplement Table 1. In addition, there was no significant correlation in the relationship between biofilm strength and ST types of the MLST (not shown).

## Discussion

Currently, there are relatively few epidemiological studies of SMA infection in Chinese children patients. In addition, a majority of these studies have been about the epidemic status with cystic fibrosis (Madi et al., 2016) and clinical characteristics or drug resistance (Hu et al., 2018; Wang et al., 2020). There is a lack of comprehensive and systematic data monitoring. Therefore, the clinical and molecular epidemic characteristics, drug resistance, virulence genes, and biofilm formation abilities of SMA infections were investigated in a children's hospital in Shanghai to provide data regarding SMA.

As immunocompromised patients, children and infants are often prone to infection with SMA. In this study, most strains were isolated from the intensive care unit, which was similar to the results of some other studies (Zhao et al., 2017; Bostanghadiri et al., 2019). This is a reminder for health care institutions to pay more attention to SMA infections in these wards. In the aspect of drug resistance, TMP/SMX is the primary clinical drug for the treatment of SMA, and drug resistance reports have gradually increased, which has aroused widespread concern (Brooke, 2012). The results of this study showed that these infections are all susceptible to levofloxacin, TMP/SMX, and minocycline, which is somewhat inconsistent with the reports of some scholars (Tokatly et al., 2019; Wang et al., 2020). It is speculated that these three drugs are not routine medicines for children and were not used before these the SMA strains were isolated. Hence, the drugs were not exposed to the SMA strains. A recent report that examined pediatric patient SMA infections in west China (Wang et al., 2020) found that TMP/SMX was still the first choice for the treatment, but the resistant rate of levofloxacin reached 40.4%. Although the resistance rate of SMA to the therapeutic drugs was low in this study, the domestic data still suggest that we still cannot relax the monitoring of drug resistance to SMA.

The protease and esterase produced by SMA can cause damage to human beings. The *stmPr1* and *stmPr2* genes can encode extracellular protease, the *smlt3773 locus* can encode outer membrane esterase, and *smf-1* is closely related to the formation of fimbriae and biofilms in cystic fibrosis (de Oliveira-Garcia et al., 2003; Nicoletti et al., 2011; DuMont et al., 2015). DuMont (DuMont et al., 2015) reported that serine protease StmPr1 and StmPr2 can affect the function of human lung epithelial cells, degrade their extracellular matrix proteins, reduce the secretion of human cytokine IL-8, and thus reduce the local immune function of the human body. In this study, the positive rates of the above four virulence genes of SMA were high, indicating that SMA may cause infection and damage related to the expression of virulence genes. In addition, two typing methods (MLST and PFGE) were used to analyze the molecular epidemic characteristics of SMA. The results showed that there was a high degree of gene polymorphism between the strains, and there was no obvious clonal transmission, which is consistent with most reports (Valdezate et al., 2004; Tanimoto, 2013; Madi et al., 2016).

This investigation found that carbapenem (81.7%) and cephalosporins (76.9%) were the primary drugs used prior to SMA infection, and the SMA biofilm formation ability was strong. The formation of biofilm has been reported to be significantly correlated with the resistance of ceftazidime, cefepime, ticacillin/clavulanic acid, piperacillin/tazobactam, aztreonam, and gentamicin (Liaw et al., 2010). Additionally, meropenem is the most common drug used in children, and ceftazidime and cefepime are also commonly used to treat other inflammation problems in children. It is highly suspected that, as a conditional pathogen, the SMA isolates were not clonally related or transmitted in this hospital, but likely the result of the drug's selectivity, which made SMA become the dominant flora and caused infection. With regard to the biofilm genes, it is worthy to note that the point mutations of the *rmlA* and *spgM* genes are different in different biofilm formation ability strains. This mutation may affect the formation of biofilm, which is also worthy of future research.

In conclusion, the genotype of the strains showed clonal diversity, and no obvious epidemic spread of SMA was found in the hospital. Most of the children had a prior history of using antibiotics, which suggested that clinical SMA infection may be an endogenous infection under drug selection. The positive rates of the virulence genes were high, and the biofilm formation ability was strong. These results are a good reminder that hospitals should use antibiotics reasonably so as to reduce the incidence of SMA infection, especially for key patient groups.

## Data Availability Statement

The raw data supporting the conclusions of this article will be made available by the authors, without undue reservation, to any qualified researcher.

## Ethics Statement

This study was approved by Ethics Committee of the Obstetrics & Gynecology Hospital of Fudan University. Written informed consent was obtained from the minor(s)' legal guardian/next of kin for the publication of any potentially identifiable images or data included in this article.

## Author Contributions

ZD performed all the experiments and wrote this manuscript. JQ assisted in the experiments operating. CL assisted in clinical information analysis. CY supervised this study. All authors approved this manuscript.

## Conflict of Interest

The authors declare that the research was conducted in the absence of any commercial or financial relationships that could be construed as a potential conflict of interest.

## Supplementary Material

Supplement Figure S1 Dendrogram of the obtained PFGE profiles of the 104 SMA strains. The distance shown in the above dendrogram represents the genetic relatedness between the analyzed strains. The isolates obtained from the patients have identical patient numbers.

Supplement Table S1 The relation of virulence genes and the ability of biofilm formation in SMA isolates. +, Gene expression in the S. maltophilia isolate; –, No gene expression in S. maltophilia.
