# Supplementary figures and images for: Clinical and Molecular Epidemiology of Stenotrophomonas maltophilia in Pediatric Patients From a Chinese Teaching Hospital
Source: Front Cell Infect Microbiol. 2020 Aug 11;10:411. doi: 10.3389/fcimb.2020.00411 (PMC7433352; doi:10.3389/fcimb.2020.00411)

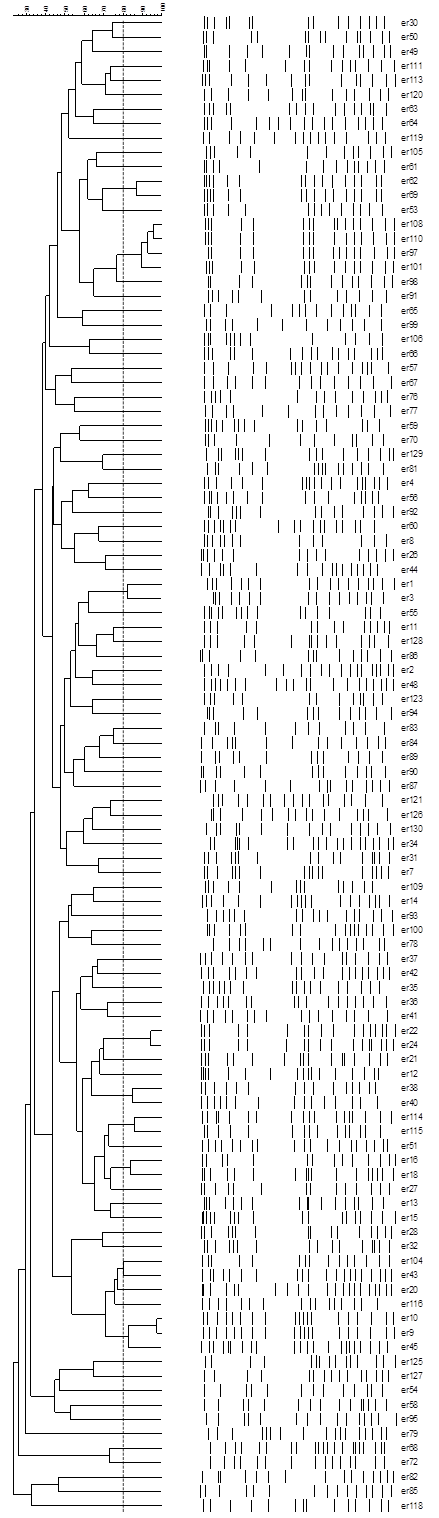

Supplement: Supplement Figure S1 — Dendrogram of the obtained PFGE profiles of the 104 SMA strains. The distance shown in the above dendrogram represents the genetic relatedness between the analyzed strains. The isolates obtained from the patients have identical patient numbers. [file Image_1.TIF]
